# Supplementary material for: Hyperinflammatory repolarisation of ovarian cancer patient macrophages by anti-tumour IgE antibody, MOv18, restricts an immunosuppressive macrophage:Treg cell interaction
Source: Nat Commun. 2025 Apr 10;16:2903. doi: 10.1038/s41467-025-57870-y (PMC11985905; doi:10.1038/s41467-025-57870-y)
Supplement: Supplementary file 1 — Supplementary Information [file 41467_2025_57870_MOESM1_ESM.pdf]

# Hyperinflammatory repolarisation of ovarian cancer patient macrophages by anti-tumour IgE antibody, MOv18, restricts an immunosuppressive macrophage:Treg cell interaction

Gabriel Osborn[1], Jacobo López-Abente[1], Rebecca Adams[1], Roman Laddach[1,2], Melanie Grandits[1], Heather J Bax[1], Jitesh Chauhan[1], Giulia Pellizzari[1], Mano Nakamura[1], Chara Stavraka[1,3], Alicia Chenoweth[1,4], Lais C G F Palhares[1], Theodore Evan[1], Jessica Hui Cheah Lim[5], Amanda Gross[6], Lenny Moise[6], Shashi Jatiani[6], Mariangela Figini[7], Rodolfo Bianchini[8], Erika Jensen-Jarolim[8,9], Sharmistha Ghosh[5], Ana Montes[5], Ahmad Sayasneh[5], Rebecca Kristeleit[3,5], Sophia Tsoka[2], James Spicer[3], Debra H Josephs[1,3], Sophia N Karagiannis[1,4*]

[1] St. John's Institute of Dermatology, School of Basic & Medical Biosciences & KHP Centre for Translational Medicine, King's College London, London, United Kingdom

[2] Department of Informatics, Faculty of Natural, Mathematical and Engineering Sciences, King's College London, Bush House, London, WC2B 4BG, UK.

[3] School of Cancer & Pharmaceutical Sciences, King's College London, Guy's Hospital, London, United Kingdom

[4] Breast Cancer Now Research Unit, School of Cancer & Pharmaceutical Sciences, King's College London, Guy's Cancer Centre, London, United Kingdom

[5] Cancer Centre at Guy's, Guy's and St Thomas' NHS Foundation Trust, London, United Kingdom

[6] SeromYx Systems, Inc, 299 Washington St, Ste D, Woburn, MA 01801, USA

[7] ANP2, Department of Advanced Diagnostics, Fondazione IRCCS, Istituto Nazionale dei Tumori, Milan, Italy

[8] Comparative Medicine, The Interuniversity Messerli Research Institute, University of Veterinary Medicine Vienna, Medical University of Vienna, University of Vienna, Vienna, Austria.

[9] Center of Pathophysiology, Infectiology and Immunology, Institute of Pathophysiology and Allergy Research, Medical University Vienna, Vienna, Austria.

*Corresponding author: Prof. Sophia N Karagiannis, St. John's Institute of Dermatology, School of Basic & Medical Biosciences & KHP Centre for Translational Medicine, King's College London, 9th Floor, Tower Wing, Guy's Hospital, London, SE1 9RT, United Kingdom

Tel: +44(0)20 7188 6355, Fax: +44(0)20 7188 8050

Email: sophia.karagiannis@kcl.ac.uk

## Supplementary Figures

**Supplementary Figure 1**

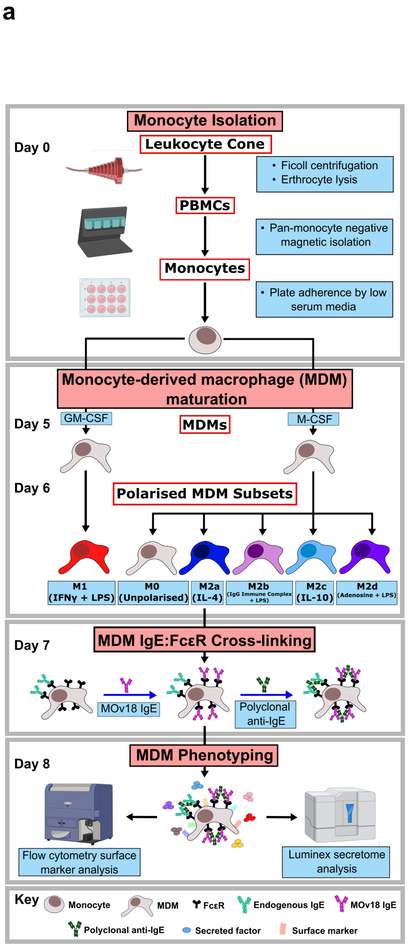


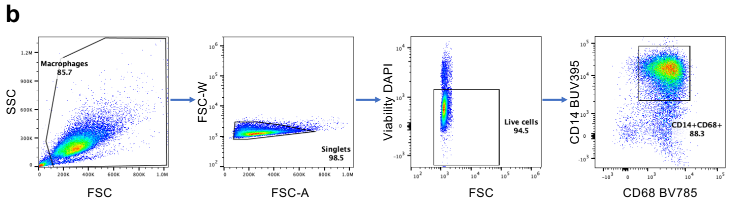


c

| Macrophage Subset | Polarisation Stimuli | Surface markers for validation | Secreted factors for validation |
|---|---|---|---|
| **M1** | IFNγ + LPS | CD40, CD80, CD86$^{high}$ CD163, MerTK$^{low}$ | IL-6, IL-12, IL-15, TNF-α, CXCL10, CXCL11$^{high}$ |
| **M2a** | IL-4 | CD200R, CD204, CD206$^{high}$ | CCL22$^{high}$ IL-6, IL-15, TNF-α$^{low}$ |
| **M2b** | IgG Immune Complex + LPS | - | IL-1β, IL-6, IL-10, TNF-α$^{high}$ |
| **M2c** | IL-10 | CD163, MerTK$^{high}$ CD40, CD80$^{low}$ | IL-10$^{high}$ IL-6, IL-15, TNF-α$^{low}$ |
| **M2d** | Adenosine + LPS | - | VEGF, CXCL10$^{high}$ |

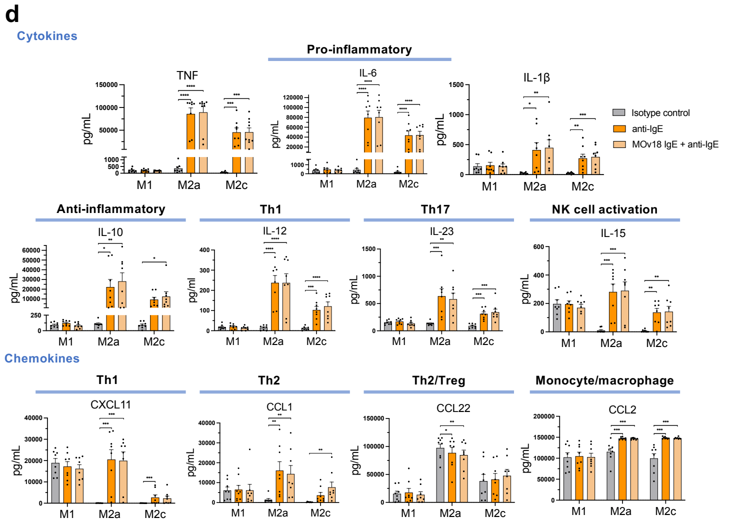


**Supplementary Fig. 1: Production of in *vitro*-derived macrophage subsets and Luminex analysis of secreted factors in macrophage culture supernatants following FcεR:IgE cross-linking.**

**(a)** Schematic of the experimental workflow for isolating monocytes from healthy volunteer leukocyte cones, maturing and polarising into *in vitro*-derived macrophage subsets (M1, M2a-d), followed by FcεR:IgE cross-linking assays by polyclonal anti-IgE antibodies. Contains images created in BioRender. Karagiannis, S. (2025) https://BioRender.com/c06z015. **(b)** Flow cytometry gating strategy for human macrophages (*in vitro*-derived macrophages, patient ascites-conditioned

macrophages (MAsc) and patient ascites isolated TAMs). **(c)** Stimuli used to polarise each of the *in vitro*-derived macrophage subsets and the surface markers and secreted factors used for their validation, measured by flow cytometry and Luminex, respectively. **(d)** Comparison of the concentrations (pg/ml) of secreted factors in the culture supernatants of *in vitro*-derived macrophage subsets following FcεR:IgE cross-linking (n=8). Data shown as mean ± SEM. Statistical significance was calculated using a repeated measures 1-way ANOVA with Tukey's post hoc test; *Padj < 0.05, **Padj < 0.01, ***Padj < 0.001 and ****Padj < 0.0001. Source data and exact P/Padj values are provided as a Source Data file.

Supplementary Figure 2

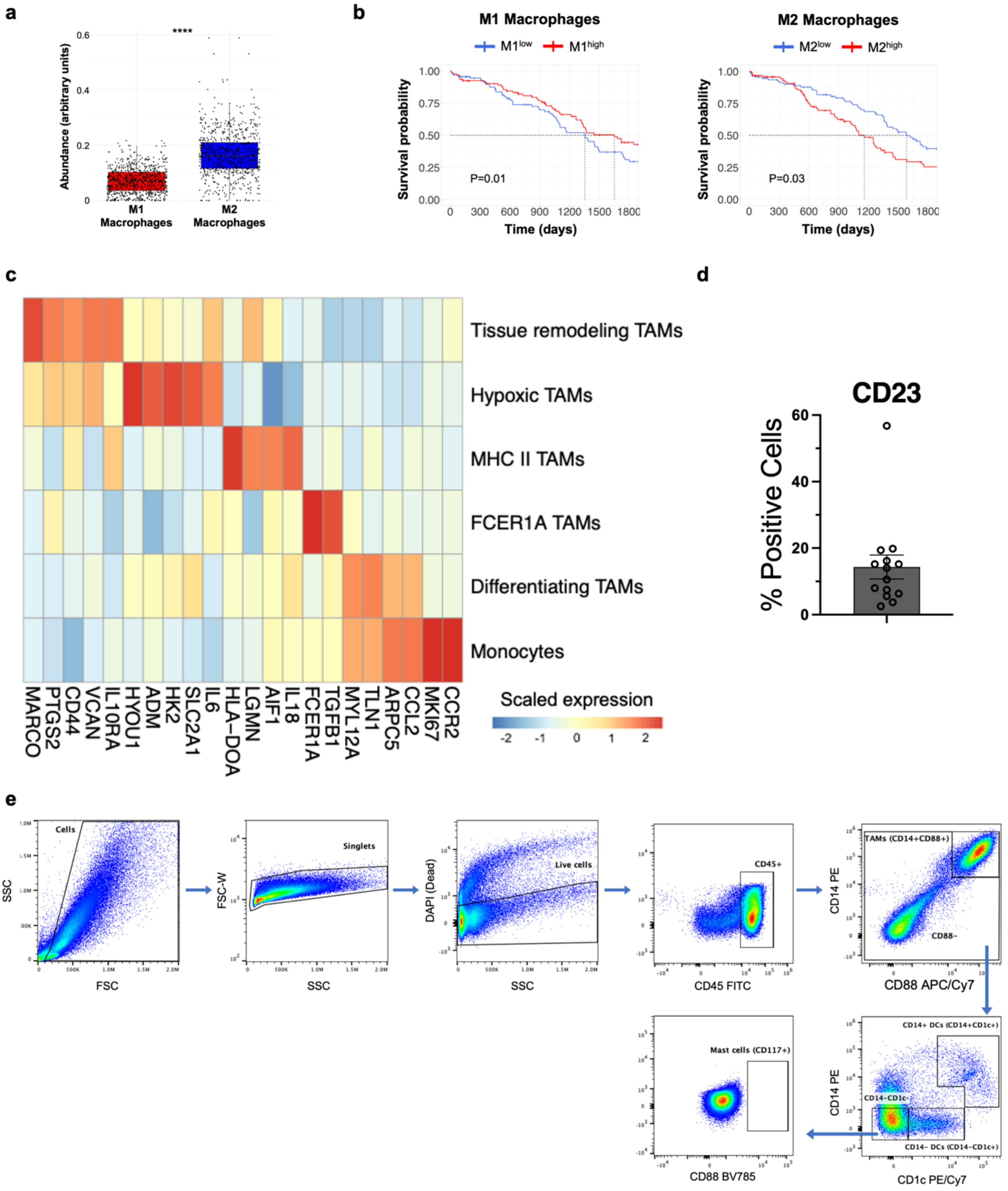


**Supplementary Fig. 2: The ovarian TME is enriched for alternatively-activated macrophages which associate with poor patient survival and express *FCER1A* and CD23.**

Bulk (TCGA-OV; n=378) and single-cell (GSE165897; n=4) RNA-seq and flow cytometric evaluation of the TME of ovarian cancer patients. **(a)** Immune cell abundance in TCGA-OV primary tumours was estimated by the CIBERTSORTx deconvolution package. Boxplot comparison of M1 and M2 macrophage abundance (n=190) (P<0.0001). **(b)** Kaplan-Meier plots stratifying TCGA-OV patients by high and low levels (quartiles) of M1 and M2 macrophage tumour abundance (Survival and Survminer packages). **(c)** Single-cell RNA-seq analysis of metastatic peritoneal tumours using the Seurat package. Monocyte and macrophage subsets were resolved by unsupervised clustering and differentially expressed genes (DEGs) between them were determined (two-tailed Wilcoxon signed-rank test). Heatmap representing the scaled expression (TPM) of DEGs used for monocyte and macrophage subset annotation. **(d)** Flow cytometric evaluation of CD23 expression on TAMs from patient ascites (n=14). **(e)** Flow cytometry gating strategy used to identify TAMs, CD14+ and CD14- dendritic cells (DCs) and mast cells in patient ascites. Data shown as median (centre line), IQR (box) and range within 1.5 x IQR (whiskers) **(a)** and mean ± SEM **(d)**. Statistical significance was calculated using a Wilcoxon signed rank test **(a)** and Flemington-Harrington weighted log rank test **(b)**; ****Padj < 0.0001. Source data and exact P/Padj values are provided as a Source Data file.

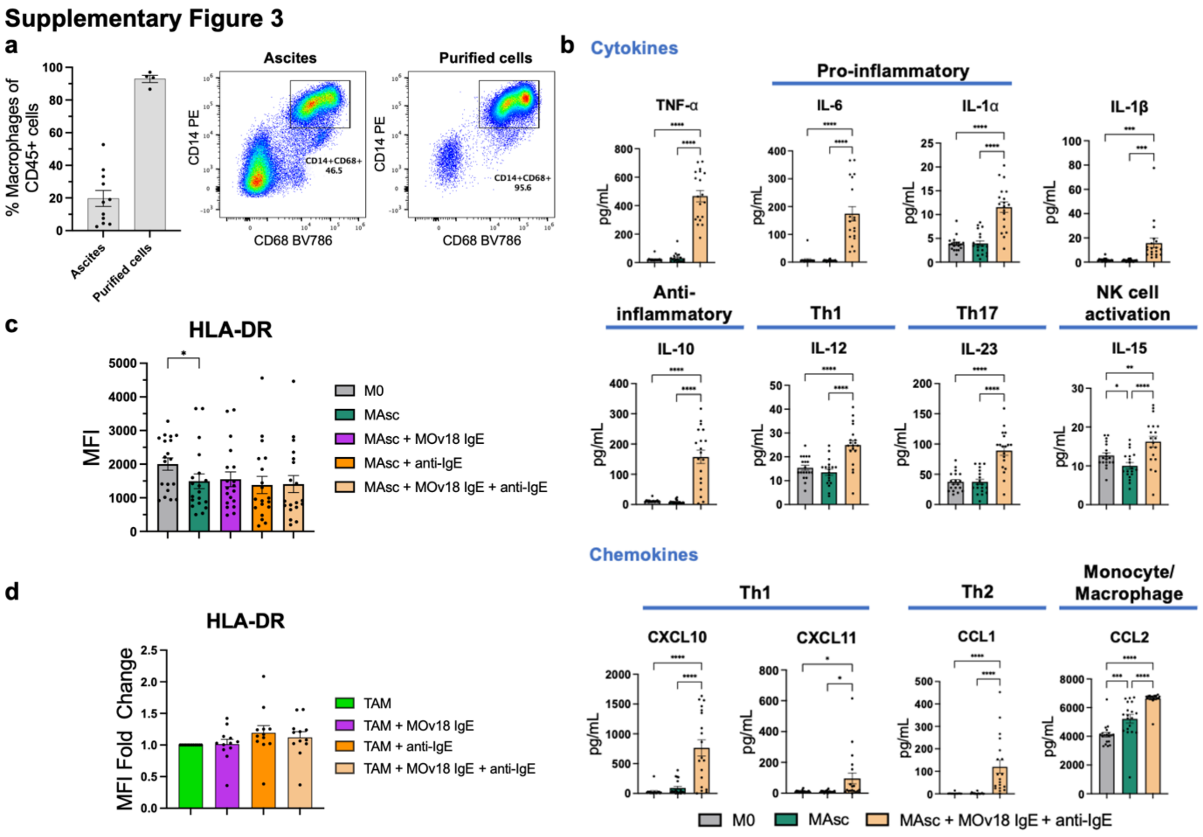


**Supplementary Fig. 3: Tumour-associated macrophages (TAMs) are abundant in ovarian cancer patient ascites and ascites-conditioned macrophages (MAsc) exhibit a broad secretome activation following *ex vivo* IgE stimulation.**

Evaluation of patient ascites TAMs and MAsc following FcεR:IgE cross-linking by polyclonal anti-IgE antibodies. **(a)** Comparison of the proportion of TAMs of CD45+ cells in patient ascites and following CD14+ magnetic isolation, with representative flow cytometry plots (n=11 ascites; n=4 purified cells). **(b)** Luminex comparison of the concentrations (pg/ml) of secreted factors in the culture supernatants of MAsc following FcεR:IgE cross-linking (n=19). **(c)** Flow cytometric evaluation of MAsc HLA-DR expression following FcεR:IgE cross-linking (n=19) (Padj=0.0296). **(d)** Flow cytometric evaluation of TAM HLA-DR expression following FcεR:IgE cross-linking (n=12). Data shown as mean ± SEM. Statistical significance was calculated using a repeated measures 1-way ANOVA with Tukey's post hoc test; *Padj < 0.05, **Padj <

0.01, ***Padj < 0.001 and ****Padj < 0.0001. Source data and exact P/Padj values are provided as a Source Data file.

**Supplementary Figure 4**

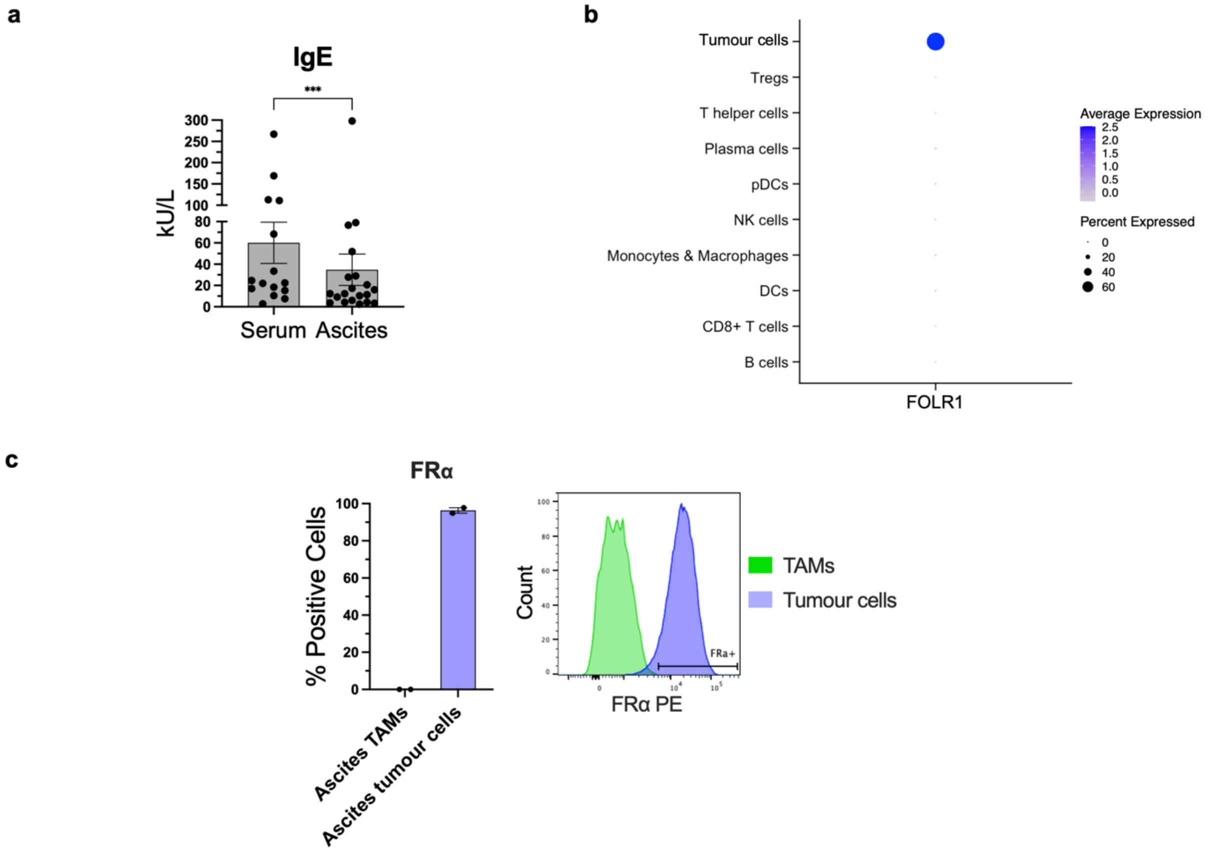


**Supplementary Fig. 4: Endogenous IgE antibodies and Folate Receptor-α (FRα)+ cancer cells are present in the tumour microenvironment (TME) of ovarian cancer patients.**

Evaluation of IgE antibody concentrations and FOLR1/FRα expression in ovarian cancer patient ascites and metastatic tumours. **(a)** Comparison of IgE concentrations in patient serum (n=15) and ascites (n=20) (P=0.0001). **(b)** scRNA-seq evaluation of FOLR1 (FRα) expression on immune cells and tumour cells in metastatic peritoneal tumours (GSE165897; n=6). **(c)** Comparison of FRα expression on tumour-associated macrophages (TAMs) and tumour cells in patient ascites, with representative flow cytometry histograms (n=2). Data shown as mean ± SEM. Statistical significance was calculated using a two-tailed Wilcoxon signed rank test; ***P < 0.001. Source data and exact P/Padj values are provided as a Source Data file.

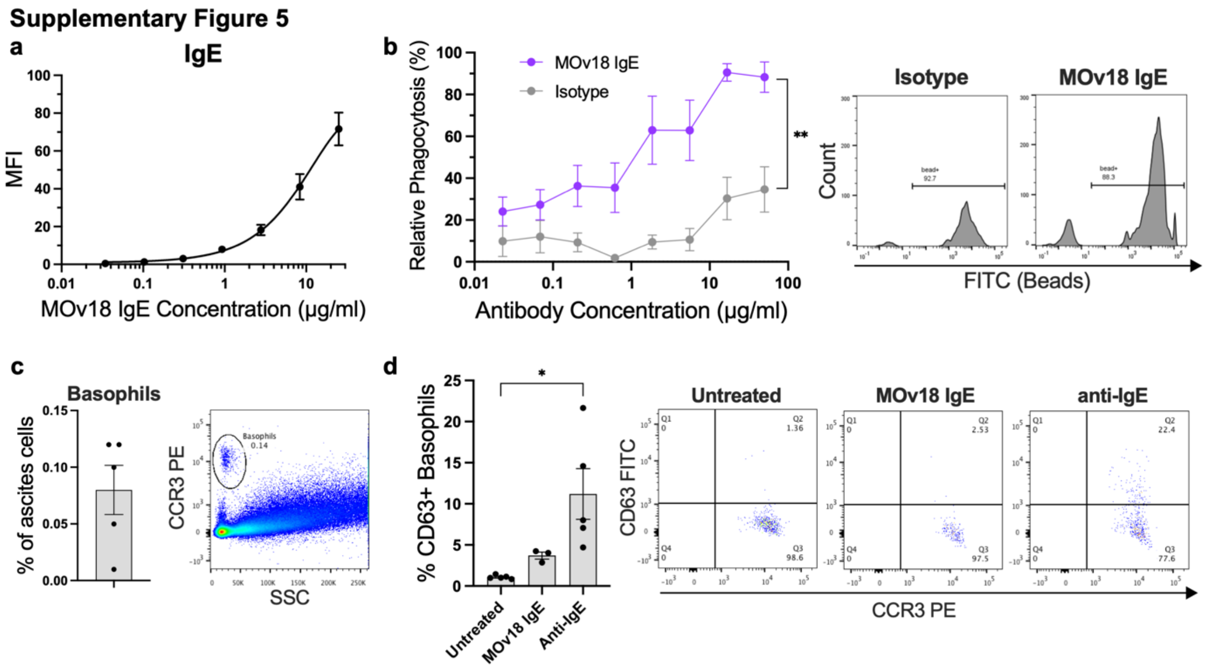


**Supplementary Fig. 5: Dendritic cells (DCs) and basophils exhibit IgE-mediated activation.**

Flow cytometric evaluation of IgE-mediated activation of human DCs and basophils. **(a)** Assessment of MOv18 IgE binding to DCs (n=4). **(b)** Comparison of DC phagocytosis of Folate Receptor-α (FRα)-coated beads following treatment with MOv18 IgE or IgE isotype control (anti-NIP IgE), with representative flow cytometry histograms (n=5) (P=0.0022). **(c)** Quantification of the proportion of basophils of total ascites cells, with a representative flow cytometry plot (n=5). **(d)** Comparison of ascites basophil activation, as measured by CD63 expression, following treatment with MOv18 IgE or polyclonal anti-IgE antibodies, with representative flow cytometry plots (n=5) (Padj=0.0213). Data shown as mean ± SEM. Statistical significance was calculated using a paired two-tailed t-test comparison of the area under the curves (AUCs) **(b)** and a repeated measures 1-way ANOVA with Tukey's post hoc test **(d)**; *Padj < 0.05, **P < 0.01. Source data and exact P/Padj values are provided as a Source Data file.

**Supplementary Figure 6**

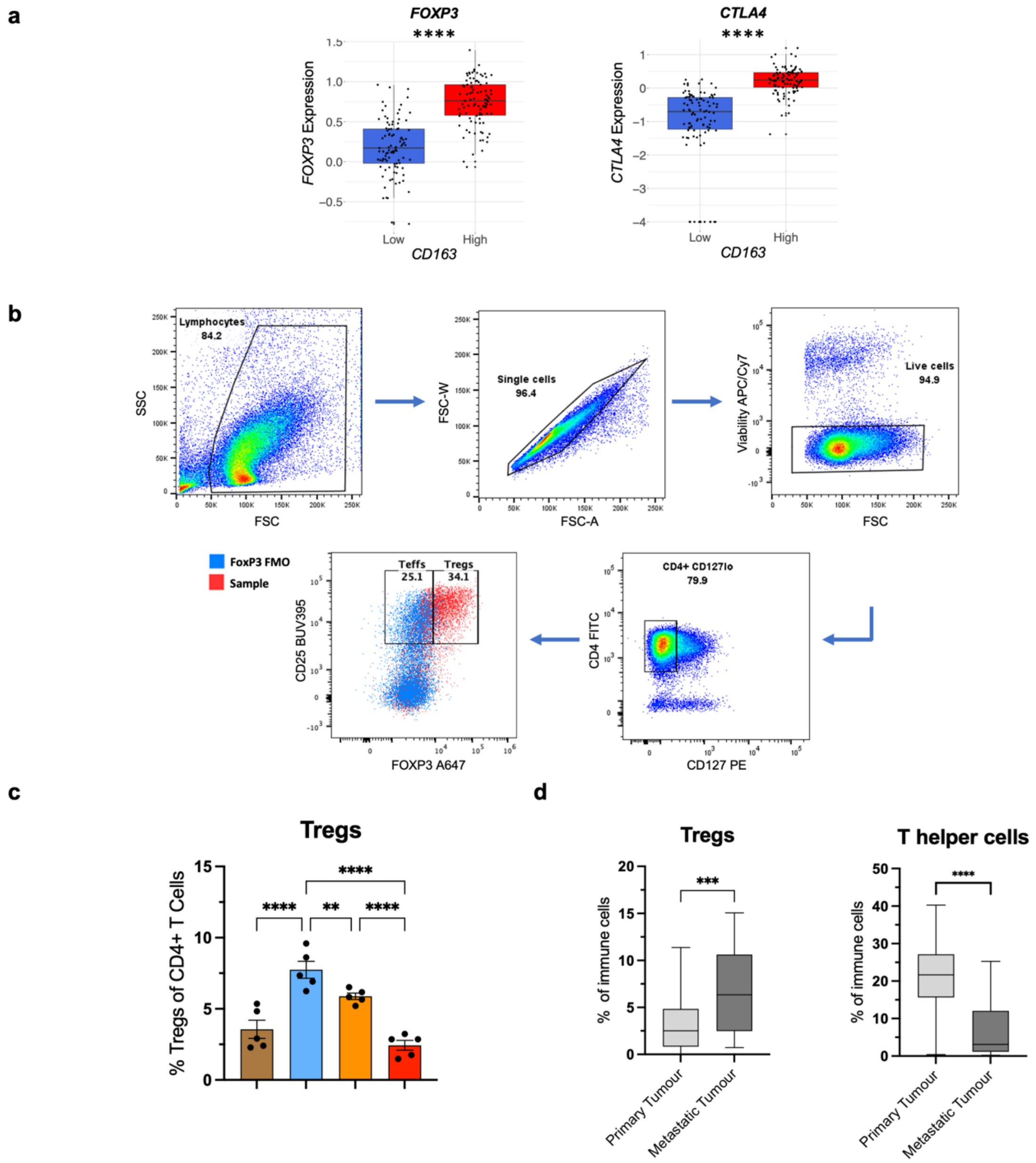


**Supplementary Fig. 6: Assessment of regulatory T cell (Treg) frequencies in primary and metastatic ovarian cancer patient tumours and *ex vivo* macrophage:naïve CD4+ T cell co-cultures.**

Evaluation of Treg frequencies in primary (TCGA-OV; n=378; bulk RNA-seq) and metastatic peritoneal (single-cell RNA-seq; GSE165897; n=6) ovarian cancer patient tumours and *ex vivo* macrophage:naïve CD4+ T cell co-cultures, following FcεR:IgE cross-linking by polyclonal anti-IgE antibodies (flow cytometry). **(a)** TCGA-OV patients

were stratified by high and low levels (quartiles) of *CD163* tumour expression. Boxplot comparison of expression ($log_{10}$(TPM+0.001)) of Treg lineage genes, *FOXP3* (P<0.0001) and *CTLA4* (P<0.0001), between *CD163*$^{high}$ and *CD163*$^{low}$ tumours (n=190). **(b)** Flow cytometry gating strategy used to gate Tregs and T effector cells (Teffs) following co-culture of macrophages (IL-10-polarised M2c or patient ascites-conditioned (MAsc)) with naïve CD4+ T cells. FMO: Fluorescence Minus One control. **(c)** M2c were co-cultured with autologous naive CD4+ T cells and the proportion of Tregs of total CD4+ cells compared (n=5). **(d)** Comparison of the proportion of Tregs (P=0.0006) and T helper cells (non-Treg CD4+ T cells) (P<0.0001) of immune cells in primary (abundance estimated by CIBERSORTx deconvolution package) and metastatic tumours (single cell RNA-seq). Data shown as median (centre line), IQR (box) and either range within 1.5 x IQR (whiskers) **(a)** or range **(d)** and mean ± SEM **(c)**. Data shown as mean ± SEM. Statistical significance was calculated using a Wilcoxon signed rank test **(a),** a repeated measures 1-way ANOVA with Tukey's post hoc test **(c)** and an unpaired two-tailed t-test **(d)**; **Padj < 0.01, ***Padj < 0.001 and ****Padj < 0.0001. Source data and exact P/Padj values are provided as a Source Data file.

## Supplementary Tables

**Supplementary Table 1** - Clinical information for the ovarian cancer patient cohort.

| Patient ID | Age at sample collection | Histology | Stage at diagnosis | Surgery prior to sample collection | Chemotherapy prior to sample collection |
|---|---|---|---|---|---|
| **4** | 79 | High grade serous ovarian cancer | III | Yes | Yes |
| **43** | 54 | High grade serous ovarian cancer | IV | Yes | Yes |
| **63** | 75 | High grade serous ovarian cancer | III | Yes | Yes |
| **76** | 74 | High grade serous peritoneal cancer | III | Yes | Yes |
| **122** | 58 | High grade serous peritoneal cancer | III | Yes | Yes |
| **123** | 75 | Low grade serous ovarian cancer | III | Yes | Yes |
| **150** | 61 | High grade serous ovarian cancer | IV | No | Yes |
| **152** | 55 | High grade serous ovarian cancer | IV | No | No |
| **153** | 56 | High grade serous ovarian cancer | IV | No | Yes |
| **154** | 44 | Borderline seromucinous ovarian cancer | I | No | No |
| **155** | 55 | Clear cell ovarian cancer | IV | No | No |
| **156** | 52 | Clear cell ovarian cancer | III | No | No |
| **157** | 56 | High grade serous ovarian cancer | III | No | No |
| **158** | 74 | High grade serous ovarian cancer | III | Yes | No |
| **162** | 65 | High grade serous fallopian tube cancer | III | No | No |
| **163** | 79 | High grade serous ovarian cancer | I | No | No |
| **164** | 69 | High grade serous ovarian cancer | III | No | No |
| **165** | 63 | High grade serous ovarian cancer | IV | No | Yes |
| **166** | 62 | High grade serous ovarian cancer | IV | No | Yes |
| **169** | 80 | High grade serous ovarian cancer | IV | No | No |
| **171** | 55 | High grade serous fallopian tube cancer | III | No | Yes |
| **173** | 54 | High grade serous ovarian cancer | IV | No | Yes |
| **174** | 56 | High grade serous ovarian cancer | IV | No | Yes |
| **178** | 45 | Mucinous ovarian cancer/cervical cancer (indistinguishable at diagnosis) | IV | No | No |
| **179** | 69 | High grade serous ovarian cancer | IV | No | Yes |
| **180** | 82 | High grade serous ovarian cancer | III | No | Yes |
| **181** | 68 | Endometroid ovarian cancer | III | No | No |
| **184** | 70 | High grade serous ovarian cancer | III | No | No |
| **185** | 54 | High grade serous ovarian cancer | IV | No | Yes |
| **187** | 42 | High grade serous ovarian cancer | IV | No | No |

**Supplementary Table 2** – Description of the experiments that clinical samples from each Patient ID were used in.

| Experiment Name | Experiment Figure Number | Number of patients | Patient IDs |
|---|---|---|---|
| Patient ascites tumour-associated macrophages (TAMs): FcεRI & CD23 expression | 2 g top, Supplementary 2d | 14 | 63, 153, 154, 155, 158, 162, 163, 166, 171, 178, 180, 181, 184, 187 |
| TAMs, dendritic cells (DCs) & mast cell frequencies in patient ascites | 2 g bottom | 11 | 123, 153, 155, 157, 158, 178, 179, 180, 181, 185, 187 |
| Patient ascites-conditioned macrophages (MAsc): phenotyping; FcεR:IgE cross-linking & FLOWSOM subset analysis | 3 a-e & Supplementary 3 b-c, 4 c-e, 5 a-d | 19 | 4, 43, 63, 76, 123, 150, 152, 153, 156, 157, 162, 163, 164, 165, 166, 169, 171, 173, 174 |
| TAMs: Phenotyping | 3 e | 10 | 63, 153, 155, 158, 162, 178, 179, 180, 181, 187 |
| MAsc: MOv18 IgE binding | 4 b | 4 | 153, 156, 157, 163 |
| TAMs: MOv18 IgE binding | 4 b | 2 | 155, 158 |
| MAsc: target-specific FcεR:MOv18 IgE cross-linking by ovarian cancer cells | 4 f | 6 | 43, 153, 157, 163, 164, 174 |
| TAMs: FcεR:IgE cross-linking | 4 g, Supplementary 3d | 13 | 63, 153, 154, 155, 158, 163, 163, 166, 178, 180, 181, 184, 187 |
| TAM: target-specific FcεR:MOv18 IgE cross-linking by ovarian cancer cells | 4 h | 5 | 150, 155, 158, 180, 181 |
| TAMs: FLOWSOM subset analysis | 5 e-g | 7 | 150, 155, 158, 166, 178, 180, 187 |
| MAsc: Naïve CD4+ T cell co-cultures | 7 a-b | 19 | 4, 43, 63, 76, 123, 150, 152, 153, 156, 157, 162, 163, 164, 165, 166, 169, 171, 173, 174 |
| MAsc: Naïve CD4+ T cell co-cultures - MAsc TGF-β expression | 7 c | 12 | 4, 43, 63, 123, 150, 152, 153, 156, 163, 164, 166, 174 |
| Patient ascites-isolated tumour-associated macrophages (TAMs): CD14+ isolation | Supplementary 3a | 4 | 153, 157, 158, 178 |

| | | | |
|---|---|---|---|
| IgE measurement in patient serum & ascites | Supplementary 4a | 20 | 4, 43, 63, 76, 123, 150, 152, 153, 156, 157, 158, 164, 165, 166, 171, 173, 174, 178, 180, 181 |
| Folate Receptor-α (FRα) expression on patient ascites TAMs and tumour cells | Supplementary 4c | 2 | 155, 180 |
| Patient ascites basophil activation test (BAT) | Supplementary 5c | 5 | 43, 63, 122, 123, 152 |

## Supplementary Methods

### Human blood cell isolation

Blood from healthy volunteer leukocyte cones was diluted with phosphate-buffered saline (PBS), layered on top of Ficoll Paque Plus and centrifuged at 1020 x g for 20 minutes at room temperature with no brake. PBMCs were harvested using a Pasteur pipette and washed with FACS buffer. Subsequently, cells were incubated in Red Blood Cell Lysis Buffer for 5 minutes at room temperature, washed and passed through a 40 *μ*m filter.

### MOv18 IgE generation

Sp2/0 hybridoma cells stably transfected with chimeric mouse/human anti-human FRα MOv18 IgE were expanded in IMDM supplemented with 10 % FBS and 2 mM L-Glutamine in a shaking incubator (37 °C, 5% $CO_2$). Cells were then transferred to a drug selection media of IMDM supplemented with 5 % FBS, 2 mM L-Glutamine and 250 μg/ml G418, 1.25 μg/ml hypoxanthine, 0.25 μg/ml mycophenolic acid, 25 μg/ml xanthine for 3 days, before expansion was resumed. Subsequently, supernatants were harvested and centrifuged at 4000 x g for 10 minutes at 4 °C, followed by sterile filtration using a 0.45 μm membrane pore size and then 0.22 μm.

MOv18 IgE was purified using CaptureSelect IgE Affinity Matrix (Thermo Fisher Scientific, 2943542005). The column was first washed and equilibrated using PBS before the cell supernatants, pre-diluted 1:1 with PBS, were run through the column. Subsequently, the column was washed with PBS, followed by elution with 20 mM citric acid (pH 3.0) into collection tubes containing 1M Tris (pH 8.0), ensuring a neutral pH

at the point of collection. MOv18 IgE was then dialysed into PBS using a Slide-A-Lyzer Dialysis Cassette (Thermo Fisher Scientific, 66454), according to the manufacturer's protocol.

Antibody purity was evaluated using SDS-PAGE electrophoresis and high-performance liquid chromatography (HPLC) and antibody antigen binding was assessed using flow cytometry.

**Binding of MOv18 IgE on macrophages**

In a round-bottom plate, a dilution series of MOv18 IgE was prepared in complete RPMI. Following detachment, macrophages were diluted to 2 x $10^6$ cells/ml and transferred to the MOv18 IgE-containing wells. Cells were then incubated for 30 minutes on ice followed by washing. Cells were then stained with an anti-human IgE FITC antibody (Vector Laboratories) and evaluated by flow cytometry. Macrophage IgE binding data are represented throughout as IgE signal above the signal from IgE endogenously-bound to macrophages following *ex vivo* culture.

**Antibody-dependent cellular cytotoxicity and phagocytosis (ADCC/ADCP) assay**

ADCC/ADCP assays were performed as described previously[1]. Briefly, IGROV1 ovarian cancer cells (target cells) were detached with trypsin and stained with CFSE (Carboxfluoroscein Succinimidyl Ester), the day prior to the experiment. The next day ascites TAMs (effector cells) were added at a 3:1 effector:target ratio to the IGROV1 cells, in the presence of 5 ug/ml MOv18 IgE or non-specific isotype control (NIP-IgE;

specific for the hapten 5-iodo-4-hydroxy-3-nitrophenyl [2]). Cells were incubated for 3 hours (37 °C, 5% $CO_2$) before evaluation by flow cytometry.

**Binding of MOv18 IgE to human dendritic cells (DCs) and DC phagocytosis assay**

Human DCs were derived by *ex vivo* maturation of human monocytes using human Mo-DC Differentiation Medium (Miltenyi, 130-094-812).

To evaluate binding to MOv18 IgE, DCs were incubated with a dilution series of fluorescently-conjugated MOv18 IgE (conjugated in-house using Alexa Fluor 488 Protein Labelling Kit (Thermo Fisher Scientific, A10235)) for 15 minutes at room temperature.

To evaluate DC phagocytosis of Folate Receptor-α (FRα)-coated beads, Carboxylate-Modified FlouSpheres beads (Invitrogen, F8823) were first coupled with recombinant FRα (Sino Biological, 11241-H08H), using the carbodiimide reagent EDC and amine-reactive Sulfo-NHS Ester (Fisher Scientific, PIA39269). MOv18 IgE or non-specific isotype control (NIP-IgE) were added to the beads in a dilution series and unbound antibody washed away. The antibody:bead complexes were added to mature DCs and incubated for 1 hour (37 °C, 5% $CO_2$).

Both the MOv18 IgE binding assay and phagocytosis assay were evaluated by flow cytometry, with DCs first gated by CD11c+. A phagocytosis score was calculated for each antibody concentration, which considers the proportion of DCs that phagocytosed beads and the degree of phagocytosis. Minimum-Maximum scaling was

then applied to these phagocytosis scores within each biological replicate, to calculate Relative Phagocytosis, as presented in the data.

**Patient ascites basophil activation test (BAT)**

100 µl of cells from ascites were incubated with 25 µl stimulation buffer (Bühlmann Laboratories AG, FK-CCR) and 50µl of MOv18 IgE or anti-IgE antibody (Agilent Dako) (3.5 µg/ml final concentration). All samples were stained with 10µl anti-CCR3-PE and anti-CD63-FITC staining cocktail (Bühlmann Laboratories AG, FK-CCR) and incubated for 30 minutes (37 °C, 5% $CO_2$). Samples were then incubated with 2ml red blood cell lysis buffer (Bühlmann Laboratories AG, , FK-CCR) for 10 minutes at room temperature. Samples were washed and resuspended in 150µl acquisition buffer (Bühlmann Laboratories AG, FK-CCR) before evaluation by flow cytometry. Basophils were gated as $CCR3^{high}$ $SSC^{low}$ and basophil activation determined by CD63 expression (Supplementary Fig. 5c-d).

**Immunohistochemical evaluation of pre- and on-treatment tumour biopsies from the Phase I clinical trial of MOv18 IgE**

The trial protocol for the Phase I clinical trial of MOv18 IgE (NCT02546921) were described previously[3]. Paired pre- and on-treatment biopsies of metastatic tumours were taken from two patients. Patients received MOv18 IgE at the second highest dose of 3 mg or the highest dose of 6 mg escalated to 12 mg.

Biopsies were fixed in 10% neutral buffered formalin (NBF) and embedded into Formalin-Fixed Paraffin-Embedded (FFPE) and then cut into 4 µm sections. Haematoxylin and eosin (H&E) or IHC staining was then completed using the Ventana

Benchmark Ultra. Tonsil sections were stained as positive and negative (no primary antibody) controls for the IHC primary antibodies.

Images were analysed in QuPath software (v0.5.1). Pixel classifiers were used to annotate tumour areas, as well as detect CD68 and CD3 expression on IHC images, and an object classifier used to annotate immune cells on H&E images. Classifiers were trained using a sparsed image, generated from regions of all images, before being applied to whole images. Intratumoural percentage for CD68 and CD3 expression and immune cells, denotes detection in the annotated tumour areas relative to detection over the whole image.

### ***In vivo*** **analysis of MOv18 IgE-treated tumours by microarray: data pre-processing**

Microarray probe intensities were background corrected, quantile normalised and summarised into expression values using the oligo R package (v1.60.0), using default settings. Probe IDs were annotated with rat gene names using the ragene10sttranscriptcluster.db R package (v8.8.0). A single mean expression value was calculated for probes which were annotated with the same rat gene name. Unannotated probes were removed. Rat gene names were converted to human gene names using the babelgene R package (v22.9). For multiple rat genes which converted to a single orthologous human gene, a single mean expression value was calculated. For a single rat gene which converted to multiple orthologous human genes, the same expression value was applied to each.

### **Analysis of patient tumours (TCGA-OV) by bulk RNA-seq: data pre-processing**

Bulk RNA-seq data, clinical information and survival information were downloaded from Xenabrowser.net and patients were only included in the analysis if all were present. Bulk RNA-seq data was downloaded as both raw counts and FPKM (Fragments Per Kilobase per Million mapped fragments). For the FPKM data, these were first converted into TPM (Transcripts Per Million). For both raw counts and TPM data, gene Ensembl IDs were replaced with gene names and duplicate gene names were assigned unique identifiers.

**Analysis of patient tumours (GSE165897) by scRNA-seq: data pre-processing and unsupervised clustering**

GSE165897 was downloaded from Gene Expression Omnibus. Using the Seurat R package (v4.3.0.1), cells were filtered to include only cells with gene counts between 200 and 3000 and mitochondrial gene reads below 7.5%, followed by log normalisation to 10000 counts per cell. Immune cells were selected (gene: *PTPRC*$^{+}$) resulting in a dataset with 25,936 genes and 7,142 cells. 500 variable genes were identified by VST, followed by scaling and principal component analysis (PCA). Unsupervised clustering was performed using the first 14 principal components (PCs) and a resolution of 0.7 and visualised by UMAP dimensionality reduction.

Monocytes and macrophages were selected. 2 patients contained an insufficient number of cells and therefore were excluded from downstream analyses involving monocytes and macrophages (cell:cell interaction, pseudotime). Monocytes and macrophages were analysed using 500 variable genes, the first 14 PCs and a clustering resolution of 0.5. One doublet cluster was removed, resulting in 6 monocyte/macrophage clusters being identified (n=1374).

## References


1. Bracher, M., Gould, H. J., Sutton, B. J., Dombrowicz, D., Karagiannis, S. N. Three-colour flow cytometric method to measure antibody-dependent tumour cell killing by cytotoxicity and phagocytosis. *Journal of Immunological Methods* **323**, 160-171 (2007).

2. Neuberger, M. S., Williams, G. T., Mitchell, E. B., Jouhal, S. S., Flanagan, J. G., Rabbitts, T. H. A hapten-specific chimaeric IgE antibody with human physiological effector function. *Nature* **314**, 268-270 (1985).

3. Spicer, J.*, et al.* Safety and anti-tumour activity of the IgE antibody MOv18 in patients with advanced solid tumours expressing folate receptor-alpha: a phase I trial. *Nature Communications* **14**, 4180 (2023).
