# Supplementary material for: Hyperinflammatory repolarisation of ovarian cancer patient macrophages by anti-tumour IgE antibody, MOv18, restricts an immunosuppressive macrophage:Treg cell interaction
Source: Nat Commun. 2025 Apr 10;16:2903. doi: 10.1038/s41467-025-57870-y (PMC11985905; doi:10.1038/s41467-025-57870-y)
Supplement: Supplementary file 2 — Reporting Summary [file 41467_2025_57870_MOESM2_ESM.pdf]

nature portfolio

Corresponding author(s): Prof Sophia Karagiannis

Last updated by author(s): Oct 26, 2024

Reporting Summary

Nature Portfolio wishes to improve the reproducibility of the work that we publish. This form provides structure for consistency and transparency in reporting. For further information on Nature Portfolio policies, see our Editorial Policies and the Editorial Policy Checklist.

Statistics

For all statistical analyses, confirm that the following items are present in the figure legend, table legend, main text, or Methods section.

n/a	Confirmed	
☐	☒	The exact sample size (*n*) for each experimental group/condition, given as a discrete number and unit of measurement
☐	☒	A statement on whether measurements were taken from distinct samples or whether the same sample was measured repeatedly
☐	☒	The statistical test(s) used AND whether they are one- or two-sided<br>*Only common tests should be described solely by name; describe more complex techniques in the Methods section.*
☐	☒	A description of all covariates tested
☐	☒	A description of any assumptions or corrections, such as tests of normality and adjustment for multiple comparisons
☐	☒	A full description of the statistical parameters including central tendency (e.g. means) or other basic estimates (e.g. regression coefficient) AND variation (e.g. standard deviation) or associated estimates of uncertainty (e.g. confidence intervals)
☐	☒	For null hypothesis testing, the test statistic (e.g. *F*, *t*, *r*) with confidence intervals, effect sizes, degrees of freedom and *P* value noted<br>*Give P values as exact values whenever suitable.*
☒	☐	For Bayesian analysis, information on the choice of priors and Markov chain Monte Carlo settings
☒	☐	For hierarchical and complex designs, identification of the appropriate level for tests and full reporting of outcomes
☒	☐	Estimates of effect sizes (e.g. Cohen's *d*, Pearson's *r*), indicating how they were calculated

Our web collection on statistics for biologists contains articles on many of the points above.

Software and code

Policy information about availability of computer code

Data collection	Flow cytometry data was collected using BD FACSDiva (v9.0.1) and Beckam Coulter CytExpert (v2.4.0.28). Luminex data was collected using xPONENT (v4.2).
Data analysis	Publicly available R packages were used to analyse rat tumour microarray data, flow cytometry data and publicly available RNA-seq datasets (TCGA-OV - bulk RNA-seq gene expression data from treatment naive ovarian cancer primary tumours; GSE165897 – single cell RNA-seq data from treatment naive ovarian cancer metastatic peritoneal tumours).<br><br>Rat tumour microarray data: microarray probe intensities were background corrected, quantile normalised and summarised into expression values using the oligo package (v1.60.0). Rat gene names were converted to human gene names using the babelgene package (v22.9). Differentially expressed genes (DEGs) between treatment and control groups were identified using the limma package (v3.54.2) and the list of all genes, ranked according to fold change, was used to calculate enrichment of gene sets within the human Gene Ontology Biological Processes (GOBP) (v2023.1) pathway database, using the fgsea package (v1.22.0). Heatmaps displaying the scaled expression of genes from selected pathways were generated using the pheatmap package (v1.0.12). Boxplot comparisons of gene expression (log2) were generated using the ggplot2 package (v3.4.4).<br><br>Immunohistochemical evaluation of pre- and on-treatment tumour biopsies from the Phase I clinical trial of MOv18 IgE: Images were analysed in QuPath software (v0.5.1). Pixel classifiers were used to annotate tumour areas, as well as detect CD68 and CD3 expression on IHC images, and an object classifier used to annotate immune cells on H&E images. Classifiers were trained using a sparsed image, generated from regions of all images, before being applied to whole images.

Flow cytometry data: All analysis was completed in FlowJo software (v10.9.0) and using the CATALYST package (v1.20.1). For CATALYST analysis (patient ascites-conditioned macrophages (MAsc) and patient ascites-isolated tumour-associated macrophages (TAMs) FcεR:IgE cross-linking), dimensionality reduction was completed using the tSNE and UMAP algorithms and macrophage subsets were identified using unsupervised clustering, via the FLOWSOM algorithm. Additionally, heatmaps displaying the scaled expression of markers (Median Fluorescence Intensity (MFI)) in each of the subsets and the mean proportion (%) of each of the subsets across the samples were generated. Heatmaps displaying the scaled expression of markers (MFI and percentage positive cells) between macrophage types (in vitro-derived subsets, MAsc and TAMs) were generated using the pheatmap package (v3.4.4).

TCGA-OV: Patients were stratified into high and low quartiles of CD163 expression (Transcripts per million (TPM)) and DEGs were identified using the DESeq2 R package (v1.36.0), and the list of all genes, ranked according to fold change, was used to calculate enrichment of gene sets within the Reactome (v2023.1) and GOBP (v2023.1) pathway databases, using the fgsea package (v1.22.0). Heatmaps displaying the scaled expression of genes (TPM) from selected pathways were generated using the pheatmap package (v1.0.12). Tumour immune cell abundance and overall tumour cell abundance (Immune Score) was estimated from the TPM matrix, using the CIBERSORTx deconvolution algorithm, via the IOBR package (v0.99.9), and ConsensusTME package (v0.0.1.9000), respectively. CIBERSORTx was used to produce a heatmap of tumour immune cell abundance. Patients were stratified into high and low tertiles or quartiles by individual gene expression (TPM) or outputs from the deconvolution analysis and Kaplan-Meier survival analysis was completed using the R packages survival (v3.5-5) and survminer (v0.4.9). Boxplot comparisons of gene expression (log10(TPM+0.001)) and tumour immune cell abundance were generated using the ggplot2 package (v3.4.4). Spearman's Rank Correlation analysis was completed between gene expression values (log10(TPM+0.001)) using the ggplot2 package (v3.4.4).

GSE165897: The Seurat package (v4.3.0.1) was used for data pre-processing and normalisation, unsupervised clustering and dimensionality reduction using the UMAP algorithm and immune cell type annotation. Seurat was additionally used to identify DEGs between monocyte and macrophage clusters, and following DEG ranking according to Padj value, the top 100 (upregulated and downregulated) DEGs were used to calculate over-representation of gene sets in the human Reactome (v2023.1) and GOBP (v2023.1) pathway databases, using g:Profiler (https://biit.cs.ut.ee/gprofiler/gost). Seurat was then used to annotate the monocyte and macrophage subsets. Receptor:ligand interactions between immune cell types were identified using the liana package (v0.1.12) and heatmap displaying interactions was produced. Pseudotime analysis was completed using slingshot package (v2.4.0), to map the differentiation trajectory of monocyte and macrophage subsets. Heatmaps displaying the scaled expression (TPM) of genes used to annotate the immune cell types and monocyte and macrophage clusters were generated using the pheatmap package (v3.4.4).

All other data representation and statistical analysis was completed in GraphPad Prism (v10).

For manuscripts utilizing custom algorithms or software that are central to the research but not yet described in published literature, software must be made available to editors and reviewers. We strongly encourage code deposition in a community repository (e.g. GitHub). See the Nature Portfolio guidelines for submitting code & software for further information.

Data

Policy information about availability of data

All manuscripts must include a data availability statement. This statement should provide the following information, where applicable:

- Accession codes, unique identifiers, or web links for publicly available datasets
- A description of any restrictions on data availability
- For clinical datasets or third party data, please ensure that the statement adheres to our policy

All data is available.
Publicly available datasets used in this study:
TCGA-OV – Xena Browser
GSE165897 – NCBI Gene Expression Omnibus, Zhang et al., Sci Adv 2022, 25;8(8):eabm1831.

Research involving human participants, their data, or biological material

Policy information about studies with human participants or human data. See also policy information about sex, gender (identity/presentation), and sexual orientation and race, ethnicity and racism.

Reporting on sex and gender	Information on patients sex was collected based on self-reporting. Female patients with ovarian cancer were recruited. Healthy volunteer leukocyte cones purchased from the UK National Health Service Blood and Transplant service (NHSBT) are anonymised, so no information on sex is provided.
Reporting on race, ethnicity, or other socially relevant groupings	N/A
Population characteristics	Adult patients over the age of 18, who were able to provide written informed consent and had histologically confirmed ovarian cancer (stages I – IV) with malignant peritoneal ascites were included. Leukocyte cones were purchased from NHSBT and derived from anonymised individuals over the age of 18, who were able to provide written informed consent.
Recruitment	Patients were identified by C.S., S.G., A.M., A.S., J.H.C.L and R.K. at Guy's and St Thomas' NHS Trust (GSTT). Adult patients with ovarian cancer with malignant peritoneal ascites were approached, otherwise no specific characteristics were used. All patient samples were collected with written informed consent.
Ethics oversight	The study was approved by the Guy's Research Ethics Committee, Guy's and St. Thomas' NHS Trust for all experiments (Reference 09/H0804/45).

Note that full information on the approval of the study protocol must also be provided in the manuscript.

Field-specific reporting

Please select the one below that is the best fit for your research. If you are not sure, read the appropriate sections before making your selection.

[x] Life sciences [ ] Behavioural & social sciences [ ] Ecological, evolutionary & environmental sciences

For a reference copy of the document with all sections, see nature.com/documents/nr-reporting-summary-flat.pdf

Life sciences study design

All studies must disclose on these points even when the disclosure is negative.

Sample size	For experiments involving patient ascites-conditioned macrophages (MAsc), due to patient heterogeneity in terms of histology, clinical stage and treatment history, we determined that a sample size of at least 15 patients was required, with 19 patient samples being used in the study. For experiments involving patient ascites-isolated TAMs, due to the need to isolate a sufficient number of TAMs for a 24-hour ex vivo culture involving 4 experimental conditions, we determined that a sample size of at least 4 patients was required, with 13 patient samples being used in the study. For ex vivo functional assays, assuming equal sample size, 80% power and a significance level of 5%, we calculated that at least 4 independent experiments were required to detect a difference between groups.
Data exclusions	No data were excluded from analyses.
Replication	In all experiments (healthy volunteer-derived and patient-derived), each sample was biologically independent. When samples were acquired in batches, we confirmed that no batch effect was present. Transcriptomic finding of an immunosuppressive TAM phenotype associated with IL10 and FCER1A expression were recapitulated ex vivo using protein-based analysis (flow cytometry) of TAMs isolated from patient ascites.
Randomization	In all experiments (healthy volunteer-derived and patient-derived), all experimental conditions were assessed for each sample.
Blinding	Blinding was applied in the immunohistochemistry evaluation of pre- and on-treatment biopsies from the Phase I trial of MOv18 IgE.

Reporting for specific materials, systems and methods

We require information from authors about some types of materials, experimental systems and methods used in many studies. Here, indicate whether each material, system or method listed is relevant to your study. If you are not sure if a list item applies to your research, read the appropriate section before selecting a response.

Materials & experimental systems

n/a	Involved in the study	
[ ]	[x]	Antibodies
[ ]	[x]	Eukaryotic cell lines
[x]	[ ]	Palaeontology and archaeology
[x]	[ ]	Animals and other organisms
[x]	[ ]	Clinical data
[x]	[ ]	Dual use research of concern
[x]	[ ]	Plants

Methods

n/a	Involved in the study	
[x]	[ ]	ChIP-seq
[ ]	[x]	Flow cytometry
[x]	[ ]	MRI-based neuroimaging

Antibodies

Antibodies used

Flow cytometry phenotyping antibodies:

Mouse Anti-Human CD86 BUV737 - clone: Clone 2331 (FUN-1) - BD Biosciences (612784)
Mouse Anti-Human CD80 BUV395 - clone: L307.4 - BD Biosciences (565210)
Mouse Anti-Human CD40 BV510 - clone: 5C3 - BioLegend (334330)
Mouse Anti-Human CD206 FITC - clone: 15-2 - BioLegend (321104)
Mouse Anti-Human CD200R PE/Dazzle 594 - clone: OX-108 - BioLegend (329310)
Mouse Anti-Human CD204 PE - clone: 7C9C20 - BioLegend (371903)
Mouse Anti-Human CD23 APC - clone: EBVCS-5 - BioLegend (338514)
Mouse Anti-Human FcεRI PE/Dazzle 594 - clone: AER-37 (CRA-1) - BioLegend (334634)
Mouse Anti-Human CD163 APC/Fire 750 - clone: GHI/61 - BioLegend (333633)
Mouse Anti-Human MerTK BV711 - clone: 590H11G1E3 - BioLegend (367619)
Mouse Anti-Human CD39 APC - clone: A1 - BioLegend (328210)
Mouse Anti-Human CD88 APC/Cy7 - clone: S5/1 – BioLegend (344324)
Mouse Anti-Human CD163 BV605 - clone: GHI/61 - BioLegend (333616)
Mouse Anti-Human CD206 BUV563 - clone: 19.2 - BD Biosciences (741398)

Mouse Anti-Human CD80 PE - clone: 2D10 - BioLegend (305208)
Mouse Anti-Human PD-L1 PerCP-Cy5.5 - clone: 29E.2A3 - BioLegend (329737)
Mouse Anti-Human HLA-DR Alexa Flour 700 - clone: L243 - BioLegend (307626)
Mouse Anti-Human FcεRI APC - clone: AER-37 (CRA-1) - BioLegend (334612)
Mouse Anti-Human CD64 FITC - clone: 10.1 - BioLegend (305005)
Mouse Anti-Human CD40 BV421 - clone: 5C3 - BioLegend (334332)
Mouse Anti-Human CD23 PE/Cy7 - clone: EBVCS-5 - BioLegend (338516)
Mouse Anti-Human CD32b PE/Dazzle 594 - clone: S18005H - BioLegend (398316)
Mouse Anti-Human CD16 BV510 - clone: 3G8 - BioLegend (302048)
Mouse Anti-Human CD14 BUV395 - clone: MϕP9 - BD Biosciences (563561)
Mouse Anti-Human CD68 BV786 - clone: Y1/82A - BioLegend (333825)
Mouse Anti-Human CD45 FITC - clone: HI30 - BioLegend (304006)
Mouse Anti-Human CD127 PE - clone: A019D5 - BioLegend (351304)
Mouse Anti-Human CD4 FITC - clone: OKT4 - BioLegend (317408)
Mouse Anti-Human CD3 FITC - clone: UCHT1 - BioLegend (300406)
Mouse Anti-Human FOXP3 Alexa Flour 647 - clone: 259D - BioLegend (320214)
Mouse Anti-Human CD25 BUV395 - clone: 2A3 - BD Biosciences (564034)
Mouse Anti-Human CD25 BV421 - clone: BC96 – BioLegend (302630)
Mouse Anti-Human TGF- β PE-CF594 - clone: TW4-9E7 - BD Biosciences (562422)
Rat Anti-Human IL-10 BV421 - clone: JES3-9D7 - BioLegend (501421)
Mouse Anti-Human TNF- α BV510 - clone: MAb11 - BioLegend (502949)
Mouse Anti-Human CD3 PE/Cy7 - clone: UCHT1 – BioLegend (300420)
Mouse Anti-Human CD3 BUV737 - clone: UCHT1 - BD Biosciences (612751)
Mouse Anti-Human CD8 BV605 - clone: SK1 - BioLegend (344741)
Mouse Anti-Human CD8 APC - clone: SK1 - BioLegend (344733)
Mouse Anti-Human CD14 PE - clone: M5E2 - BioLegend (301806)
Mouse Anti-Human CD1c PE/Cy7 - clone: L161 - BioLegend (331516)
Mouse Anti-Human CD117 BV785 - clone: 104D2 - BioLegend (313238)
Mouse Anti-Human FcεRI PE - clone: AER-37 (CRA-1) - BioLegend (334610)
Mouse Anti-Human CD88 APC - clone: S5/1 - BioLegend (344310)

MOv18 IgE clinical trial tumour biopsy IHC antibodies:

Rabbit anti-Human CD3 - clone (2GV6) - Roche (05278422001 (790-4341))
Mouse anti-Human CD68 - clone KP-1 - Roche (790-2931)

Polyclonal Goat Anti-Human IgE Epsilon Chain, Fluorescein - Vector Laboratories (FI-3040-.5)

Polyclonal Goat Anti-Human IgE Epsilon Chain - Abcam (ab9159)

Validation

All flow cytometry phenotyping antibodies were validated by the manufacturer: BioLegend and BD Biosciences. All antibodies were titrated against the relevant cell type before being utilised in experiments. To ensure correct gating of cells which expressed the marker the antibodies were specific for, for lowly expressed markers, isotype controls were used, and in multi-fluorophore panels, Fluorescence Minus One (FMO) controls were used.

Polyclonal Goat Anti-Human IgE, Epsilon Chain Specific, Fluorescein - Vector Laboratories (FI-3040-.5): This antibody was validated by the manufacturer: "The goat anti-human Ig antibodies are prepared by hyperimmunizing animals in a manner that produces high affinity antibodies. These are then purified by an affinity chromatography procedure designed to remove any low affinity antibodies which may be present. Cross-reactivities that are likely to interfere with specific labelling are removed by solid-phase adsorption techniques. The final product is then subjected to rigorous quality control assays including immunodiffusion, solid-phase enzyme immunoassays, gel electrophoresis and solid-phase binding assays. In preparing the labelled antibodies, great care is taken to ensure the maximum degree of labelling with no alteration in the specificity and affinity of the antibody. The labelled antibody then undergoes a further series of quality control assays, including immunohistochemical analysis. This chain-specific antibody is produced specifically to distinguish between chains or classes of target immunoglobulins. This chain specific antibody has virtually no cross-reactivity with other immunoglobulin classes or other heavy or light chains." To ensure correct flow cytometric gating of IgE+ cells, an isotype control was used. (McCraw et al., 2022; Pellizzari et al., 2019; van Erp et al., 2018; Greer et al., 2014).

Polyclonal Goat Anti-Human IgE - Abcam (ab9159): This antibody was validated by the manufacturer: "Goats were immunized with a mixture of several highly purified human IgE myeloma proteins in Freund's adjuvant. Serum was collected and passed through several non-IgE containing immunoaffinity supports to make the preparation Epsilon chain specific. The anti Epsilon was immunoaffinity purified off an immunoabsorbant containing a different IgE than was used for immunization thus minimizing idiotypic influences. The affinity purified anti IgE reacts only with IgE (several different IgE kappa and lambda myeloma proteins) by Immunoelectrophoresis (IEP) and gel diffusion techniques." To ensure target specificity in functional assays, an isotype control was used. (Chauhan et al., 2023; Malpica et al., 2019; Zhang et al., 2019; Vanshylla et al., 2018; Zhang et al., 2017).

MOv18 IgE clinical trial tumour biopsy IHC antibodies. All antibodies are part of the Roche catalogue and are optimised for use on the Ventana Benchmark system. In our experiments, tonsil sections known to express the markers, were stained as positive and negative (no primary antibody) controls.

Eukaryotic cell lines

Policy information about cell lines and Sex and Gender in Research

Cell line source(s)	IGROV1 (CVCL 1304) - Sigma Aldrich (SCC203)
Authentication	Cell lines were originally obtained from a commercial source and authenticated using short tandem repeat DNA profiling.
Mycoplasma contamination	The cells were regularly tested for mycoplasma contamination and we confirm that they were negative for mycoplasma.
Commonly misidentified lines (See ICLAC register)	None.

Plants

Seed stocks	*Report on the source of all seed stocks or other plant material used. If applicable, state the seed stock centre and catalogue number. If plant specimens were collected from the field, describe the collection location, date and sampling procedures.*
Novel plant genotypes	*Describe the methods by which all novel plant genotypes were produced. This includes those generated by transgenic approaches, gene editing, chemical/radiation-based mutagenesis and hybridization. For transgenic lines, describe the transformation method, the number of independent lines analyzed and the generation upon which experiments were performed. For gene-edited lines, describe the editor used, the endogenous sequence targeted for editing, the targeting guide RNA sequence (if applicable) and how the editor was applied.*
Authentication	*Describe any authentication procedures for each seed stock used or novel genotype generated. Describe any experiments used to assess the effect of a mutation and, where applicable, how potential secondary effects (e.g. second site T-DNA insertions, mosiacism, off-target gene editing) were examined.*

Flow Cytometry

Plots

Confirm that:

- [x] The axis labels state the marker and fluorochrome used (e.g. CD4-FITC).
- [x] The axis scales are clearly visible. Include numbers along axes only for bottom left plot of group (a 'group' is an analysis of identical markers).
- [x] All plots are contour plots with outliers or pseudocolor plots.
- [x] A numerical value for number of cells or percentage (with statistics) is provided.

Methodology

Sample preparation

Patient ascites sample processing and cell isolation:

Ovarian cancer patient peritoneal ascites samples were aseptically collected and processed immediately. Ascitic fluid was first passed through a 100 µm filter and centrifuged at 490 x g for 15 minutes at room temperature. Red blood cells were lysed for 5 minutes at room temperature and then washed. Cells were then incubated with Accutase for 10 minutes at room temperatures, to ensure dissociation of multi-cellular structures, before being passed through a 70 µm filter and washed. For mononuclear cell isolation, standard Ficoll separation was then completed. For the isolation of TAMs, CD14+ cells were isolated from ascites mononuclear cells using CD14 MicroBeads (Miltenyi), according to the manufacturer's protocol. Cells were then plated on flat-bottom cell culture plates in Macrophage Attachment Media (RPMI 1640 with 2 % heat-inactivated fetal bovine serum (FBS), 2 mM L-Glutamine and 100 U/ml Penicillin-Streptomycin) at 1 million cells/ml. After a 2-hour incubation (37 °C, 5% CO2), the non-adherent cells were washed off and the media replaced with complete RPMI (RPMI 1640 with 2 % FBS, 2 mM L-Glutamine and 100 U/ml Penicillin-Streptomycin).

MOv18 IgE clinical trial metastatic tumour biopsies were fixed in 10% neutral buffered formalin (NBF) and embedded into Formalin-Fixed Paraffin-Embedded (FFPE) and then cut into 4 µm sections. Haematoxylin and eosin (H&E) or IHC staining was then completed using the Ventana Benchmark Ultra.

Human monocyte isolation from healthy volunteer leukocyte cones and ex vivo macrophage derivation:

Monocyte isolation - Following isolation of peripheral blood mononuclear cells (PBMCs) by standard Ficoll separation from leukocyte cones, monocytes were isolated by negative selection using the Pan Monocyte Isolation Kit (Miltenyi), according to the manufacturer's protocol. Monocytes were then attached to flat-bottom cell culture plates using the same protocol as described above for TAM attachment;

Macrophage derivation and polarisation - For derivation of both in vitro-derived macrophage subsets (M0, M1, M2a-d) and MAsc, following attachment of human monocytes, complete RPMI was added, supplemented with 50 ng/ml GM-CSF (M1) or 50 ng/ml M-CSF (M0, M2a-d, MAsc) (PeproTech) (Supplementary Fig. 1a). Additionally, for MAsc, media contained 10 % cell-free ascites fluid from an individual patient (Fig. 3a). Monocytes were then incubated at 37 °C, 5% CO2. On day 3, half of the

media was replaced with complete RPMI supplemented with GM-CSF or M-CSF (50 ng/ml). For MAsc, media contained 10 % cell-free ascites fluid from the same patient. MAsc derivation was complete on day 6 (Fig. 3a). To polarise macrophages to the in vitro-derived subsets, on day 5 mature macrophages were washed and incubated for 24 hours in complete RPMI with GM-CSF or M-CSF (50 ng/ml), supplemented with the following polarisation stimuli (Supplementary Fig. 1a). M0: unstimulated; M1: IFN (Thermo Fisher Scientific) (20 ng/ml) + LPS (Sigma-Aldrich) (100 ng/ml); M2a: IL-4 (PeproTech) (20 ng/ml); M2b: 20 μg/ml plate-bound anti-NIP ((4-hydroxy-3-iodo-5-nitrophenyl)acetic acid) IgG1 (generated in-house) + LPS (100 ng/ml); M2c: IL-10 (PeproTech) (20 ng/ml); M2d: 5'-N-Ethylcarboxamidoadenosine (NECA) (Cambridge Bioscience) (1.5 μg/ml) + LPS (100 ng/ml).

Detachment of macrophages (in vitro-derived subsets, MAsc and TAMs) prior to flow cytometry staining was completed using Accutase.

Co-culture of macrophages with naïve CD4+ T cells:

Co-culture experimental set-up - PBMCs from an allogeneic leukocyte cone were thawed, followed by isolation of naïve CD4+ T cells using the Naïve CD4+ T Cell Isolation Kit II (Miltenyi), according to the manufacturer's protocol. Isolated naïve CD4+ T cells were resuspended at 5 x 105 cells/ml in complete RPMI containing T cell TransAct (Miltenyi), a CD3/CD28 agonist, at a 1:1000 dilution. Derived macrophages were washed and naïve CD4+ T cells added to the macrophage cultures. Cells were co-cultured for 72 hours (37 °C, 5% CO2). At the completion of the co-cultures, PBS supplemented with 2 mM EDTA was added to ensure detachment of any semi-adherent T cells;

Intracellular cytokine staining following co-culture experiments – Co-culture cells were resuspended at 1 x 106 cells/ml in T cell Media (RPMI 1640 supplemented with 10 % heat inactivated Human AB serum, 2 mM L-Glutamine and 100 U/ml Penicillin-Streptomycin) supplemented with an activation cocktail comprising 50 ng/ml PMA (Phorbol 12-myristate 13-acetate) (Sigma-Aldrich), 1 μg/ml Ionomycin (Sigma-Aldrich) and 5 μg/ml Brefeldin A (Sigma-Aldrich)). Cells were then incubated for 5 hours (37 °C, 5% CO2) followed by flow cytometry staining;

PBMC Suppression Assay following co-culture experiments - 60 hours after the initiation of co-cultures, PBMCs from an allogeneic leukocyte cone were thawed, resuspended in complete RPMI supplemented with 60 U/ml IL-2 (PeproTech) and incubated (37°C, 5% CO2) overnight. At the completion of the co-culture (72 hours), these PBMCs were stained with CellTrace Violet (Thermo Fisher Scientific), according to the manufacturer's protocol. CellTrace dyes facilitate analysis of cellular proliferation; upon cell division the intensity of cell staining is reduced. From the co-culture wells, CD25+ T cells (Regulatory T cells (Tregs) and T effector cells (Teffs)) were isolated using CD25 MicroBeads II (Miltenyi), according to the manufacturer's protocol. The isolated CD25+ T cells were resuspended at 1 x 106 cells/ml in T cell media supplemented with 500 U/ml IL-2 and transferred to a round bottom plate in 1:2 serial dilutions. The Cell Trace Violet-stained PBMCs were resuspended at 1 x 106 cells/ml in T cell Media supplemented with 500 U/ml IL-2 and T cell TransAct at a 1:400 dilution and transferred to the plate containing CD25+ T cells in serial dilutions. This resulted in a dilution series ratio of CD25+ T cells:PBMCs ranging from 1:1 to 1:32. Positive and negative control wells were prepared in triplicate, comprising PBMCs only, with and without TransAct, respectively. Cells were then incubated (37 °C, 5% CO2) for 96 hours followed by flow cytometry staining.

Flow cytometry staining:

Cells were placed in a round-bottom 96-well plate and washed with fluorescence-activated cell sorting (FACS) buffer (PBS supplemented with 2% FBS and 2mM EDTA). Cells were then resuspended in FACS buffer supplemented with pre-titrated fluorescently-conjugated antibodies and Fc block and incubated on ice for 30 minutes. For flow cytometry marker panels that required intracellular marker staining, the Foxp3/Transcription Factor Staining Buffer Set (eBioscience) was used, according to the manufacturer's protocol. In experiments that utilised flow cytometry panels containing more than 1 Brilliant Violet fluorophore, half of the FACS Buffer volume in the staining solution was replaced with BD Horizon Brilliant Stain Buffer. To ensure correct gating of cells expressing the marker the flow cytometry antibodies were specific for, for lowly expressed markers isotype controls were used and in multi-fluorophore panels, Fluorescence Minus One (FMO) controls were used.

Instrument

Samples were acquired using a BD Fortessa, BD FACSCanto II or Beckman Coulter CytoFLEX.

Software

BD FACSDiva (v9.0.1) was used on the BD Fortessa and FACSCanto II and Beckam Coulter CytExpert (v2.4.0.28) was used on the Beckman Coulter CytoFLEX. All analysis was completed in FlowJo software (v10.9.0) and using the CATALYST package (v1.20.1).

Cell population abundance

Patient ascites-isolated TAMs: TAMs were isolated from ovarian cancer patient ascites mononuclear cells using CD14 MicroBeads (Miltenyi). TAMs (CD45+CD14+CD68+) represented ~ 20 % of CD45+ cells in ascites (Supplementary Fig. 3a). Purity of isolated TAMs of CD45+ cells ~ 93 % (Supplementary Fig. 3a). After TAM attachment and functional assays, followed by Accutase detachment, TAM purity was ~ 97 %.
Additional patient ascites immune cells: CD14+ DCs (CD45+CD14+CD88-CD1c+) represented ~ 2% of CD45+ cells (Fig. 3g); CD14- DCs (CD45+CD14-CD88-CD1c+) represented ~ 3% of CD45+ cells (Fig. 3g); mast cells (CD45+CD14-CD88-CD1c-CD117+) were absent (Fig. 3g); and basophils (CCR3high SSClow) represented ~ 0.1 % of total cells (Supplementary Fig. 5c).
In vitro-derived macrophage subsets and MAsc: Monocytes were isolated from healthy volunteer leukocyte cone PBMCs by negative selection using the Pan Monocyte Isolation Kit (Miltenyi). Purity of isolated monocytes, using CD14 and CD16, was ~ 96 %. After monocyte attachment and macrophage derivation, followed by Accutase detachment, macrophage purity (CD14 +CD68+) was ~ 92 % (Supplementary Fig. 1b).
Co-culture of macrophages with naïve CD4+ T cells: PBMCs from a healthy volunteer leukocyte cone were thawed, followed by isolation of naïve CD4+ T cells (CD4+CD45RA+) using the Naïve CD4+ T Cell Isolation Kit II (Miltenyi). Purity of isolated naïve CD4+ T cells was ~ 96%. At the completion of the co-cultures, PBS supplemented with 2 mM EDTA was added to ensure detachment of any semi-adherent T cells. Following detachment, in control conditions, Tregs and Teffs (CD4+ CD127low CD25hiFOXP3+ (Tregs) and CD25hiFOXP3- (Teffs)) (Supplementary Fig. 6b) each represented 12 % - 25 % of CD4+ T cells (Fig.

6-7). CD4+CD25+ T cells (Tregs and Teffs) were isolated from the co-cultures, at their completion, using CD25 MicroBeads II (Miltenyi). The purity of isolated CD4+CD25+ T cells was ~ 91 %.

Gating strategy	Macrophages (in vitro-derived macrophages, MAsc and TAMs) in ex vivo assays were gated by selecting live, singlet, CD14+ CD68+ cells (Supplementary Fig.1b).<br>Patient ascites cells (Supplementary Fig. 2e & 5c): macrophages (CD45+CD14+CD88+); CD14+ DCs (CD45+CD14+CD88-CD1c+); CD14- DCs (CD45+CD14-CD88-CD1c+); mast cells (CD45+CD14-CD88-CD1c-CD117+); basophils (CCR3high SSClow) and tumour cells (EpCAM+)<br>Following the completion of the macrophage:CD4+ T cell co-cultures, Tregs and Teffs were gated by selecting live, singlet, CD4+ CD127low cells, followed by CD25hiFOXP3+ (Tregs) and CD25hiFOXP3- (Teffs) (Supplementary Fig. 6b).

☒ Tick this box to confirm that a figure exemplifying the gating strategy is provided in the Supplementary Information.
